# A Rapid Screening Assay to Search for Phosphorylated Proteins in Tissue Extracts

**DOI:** 10.1371/journal.pone.0050025

**Published:** 2012-11-15

**Authors:** Ignazio Garaguso, Juergen Borlak

**Affiliations:** Centre for Pharmacology and Toxicology, Hannover Medical School, Hannover, Germany; Ottawa Hospital Research Institute, Canada

## Abstract

Reversible protein phosphorylation is an essential mechanism in the regulation of diverse biological processes, nonetheless is frequently altered in disease. As most phosphoproteome studies are based on optimized *in-vitro* cell culture studies new methods are in need to improve *de novo* identification and characterization of phosphoproteins in extracts from tissues. Here, we describe a rapid and reliable method for the detection of phosphoproteins in tissue extract based on an experimental strategy that employs 1D and 2D SDS PAGE, Western immunoblotting of phosphoproteins, in-gel protease digestion and enrichment of phosphorpeptides using metal oxide affinity chromatography (MOAC). Subsequently, phosphoproteins are identified by MALDI-TOF-MS/MS with the CHCA-TL or DHB ML sample matrix preparation method and further characterized by various bioinformatic software tools to search for candidate kinases and phosphorylation-dependent binding motifs. The method was applied to mouse lung tissue extracts and resulted in an identification of 160 unique phosphoproteins. Notably, TiO_2_ enrichment of pulmonary protein extracts resulted in an identification of additional 17 phosphoproteins and 20 phosphorylation sites. By use of MOAC, new phosphorylation sites were identified as evidenced for the advanced glycosylation end product-specific receptor. So far this protein was unknown to be phosphorylated in lung tissue of mice. Overall the developed methodology allowed efficient and rapid screening of phosphorylated proteins and can be employed as a general experimental strategy for an identification of phosphoproteins in tissue extracts.

## Introduction

Reversible protein phosphorylation is a major cellular mechanism in the regulation of protein function and activity. Such post-translational modifications of proteins are accomplished by the activities of protein kinases and reversed by phosphatases in a highly dynamic manner. Approximately 500 protein kinases are encoded by the human genome to possibly phosphorylate more than 100 000 sites [1]. The predominant class of protein phosphorylation in eukaryotic cells is O-phosphates, and modifications occur on serine (S), threonine (T) and tyrosine (Y) residues at a stoichiometric ratio of 86.4%, 11.8% and 1.8%, respectively [2]. Phosphorylation of proteins changes their activities and is associated with translocation and modulation of protein–protein interaction to influence cellular processes including signal transduction, cell differentiation, proliferation, metabolic maintenance, cell division, as well as programmed cell death [3]. Importantly, an imbalance between phosphorylation and de-phosphorylation results in a wide range of pathological conditions. Thus, inhibiting kinases is the subject of molecular targeted therapies, particularly in the treatment of cancers where hyperactivity of kinases is frequently observed [4]–[7].

While it is highly desirable to study entire phosphoproteomes, an identification of low abundance phosphoproteins and an assessment of the stoichiometry of protein phosphorylation is challenging, especially when current protocols are applied to small amounts of tissues such as that of lung of mice. As a result of this only a small number of investigators reported studies on the pulmonary proteome and these are based primarily on cell lines [8]–[10] or nasal/bronchoalveolar lavage [11]–[13]. However, in biomedical research diverse mouse models are employed to study disease mechanisms, yet the mapping of components, regulatory events and substrates in signaling pathways remains challenging and is impaired by the lack of an easy method to study more comprehensively entire proteomes. Nonetheless, in recent years, research on phosphoproteins benefitted from the availability of antibodies that selectively recognize phosphorylated amino acid residues [14]–[17], thus enabling a more broad search of phosphoproteins [18] even though some may prove to be ineffective in the recognition of phosphoproteins [14]. Thus, a variety of experimental strategies for the enrichment and detection of phosphorylated proteins has been developed but none of these approaches can be regarded as universally applicable with the mapping and characterization of phosphoproteins requiring a combination of different methods and experimental strategies [19]. Specifically, metal oxide affinity chromatography (MOAC) with titanium dioxide (TiO_2_) has been employed for the selective enrichment of phosphopeptides prior to MS [20], [21]. This technique is based on the selective interaction of phosphopeptides with porous TiO_2_ microspheres (titanspheres) via bidentate binding at the TiO_2_ surface and in combination with MALDI-MS to allow the detection of phosphopeptides. Likewise, separation of complex protein mixtures by two-dimensional electrophoresis (2-DE) and the combination of 2-DE with MALDI-MS for proteome and phosphoproteome mapping studies have been reported as a successful strategy [22]–[24] while other investigators used HPLC- coupled with ESI-MS/MS.

**Figure 1:**
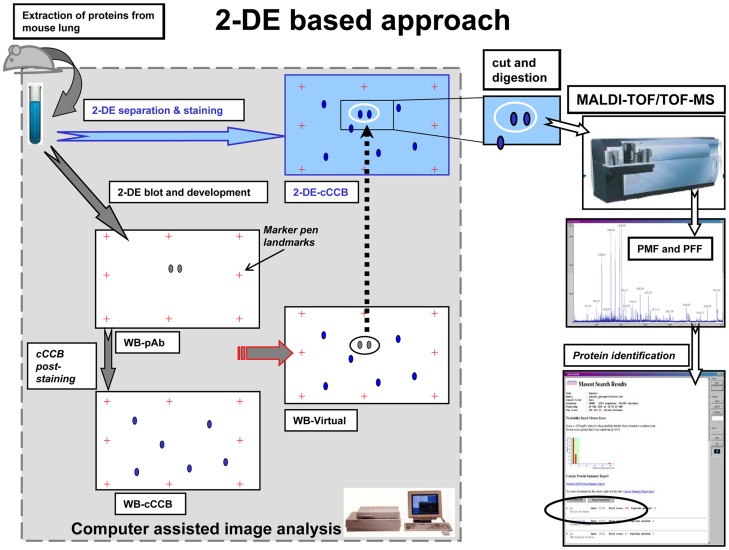
Description of the combined 2-DE-WB approach. 200 µg of tissue lysate protein extracts were separated on 2-DE, stained with Colloidal Coomassie G-250 and acquired as an image (2-DE-cCBB). In parallel 40 µg of total protein extract was separated by 2-DE and subsequently transferred onto PVDF membrane for incubation with antibodies directed against phosphorylated proteins. With a marker pen, landmarks points (crosses in Figure) were set around the membrane and the image of phosphorylated proteins was acquired (WB-pAb). Subsequently, the total proteins on the same membrane were revealed by cCCB-post staining and the image was recorded (WB-CBB). Using the marker added landmarks the two images were superimposed and combined by the image analysis software to create a virtual image showing the phosphoproteins and the total proteins together (WB-virtual). Several protein spots from this image, which are in common with the cCBB-2-DE image, were selected as additional landmarks and used to superimpose the WB-CCB image to the 2-DE-cCBB image. This allowed deciphering of phosphorylated proteins on the gel. Highlighted spots were then excised from the gel using a spot cutter, followed by in-gel digestion using trypsin. The proteins were identified using MALDI-TOF MS.

To overcome current limitations in phosphoproteomic analyses of tissue extracts a simple and fast method was developed consisting of 1D or 2D SDS-PAGE, Western immunoblotting (WB) of phosphoproteins, in-gel protease digestion and in the case of 1D gel electrophoresis enrichment of phosphopeptides using TiO_2_-MOAC micro columns. Subsequently, proteins were analyzed by MALDI-TOF-MS/MS and further characterized using various bioinformatic software tools to search for candidate kinases and phosphorylation-dependent binding motifs. Finally, phosphoproteins were classified with respect to their Gene Ontology (GO).

**Figure 2:**
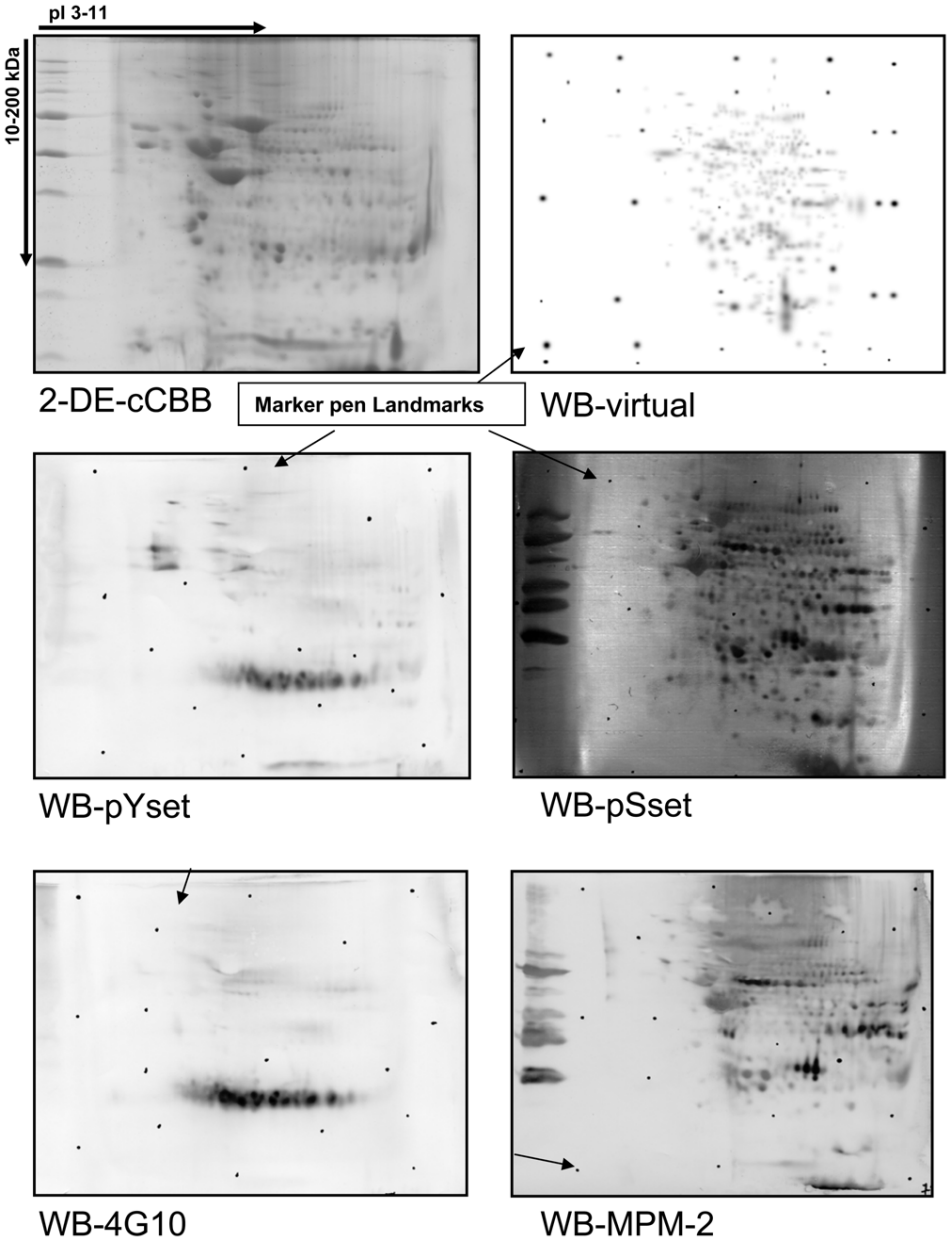
2-DE maps. 200 µg of tissue lysate protein extracts were separated on 2-DE and stained with Colloidal Coomassie G-250 and acquired as an image (2-DE-cCBB). In parallel 40 µg of total protein extract was separated by 2-DE and subsequently transferred onto PVDF membrane for incubation with antibodies directed against phosphorylated proteins. With a marker pen, landmarks points (some are highlighted by arrows in the Figure) were set around the membrane to be used for computer assisted merging and matching of images of phosphorylated proteins as acquired with the Abs WB-Yset, WB-pSset, WB-4G10, WB-MPM-2. Subsequently, protein spots on the same membrane were revealed by cCCB-post staining and the image was recorded (not shown). Using the marker added landmarks, the images were superimposed and combined by the image analysis software to create a virtual image showing the phosphoproteins and the total proteins together (WB-virtual).

## Materials and Methods

### Ethics Statement

All animal work followed strictly the Public Health Service (PHS) Policy on Humane Care and Use of Laboratory Animals. Formal approval to carry out animal studies was granted by the institutional board and the ethical review board of the city of Hannover, Germany. The approval ID is Az: 33.9-42502-04-06/1204.

**Figure 3:**
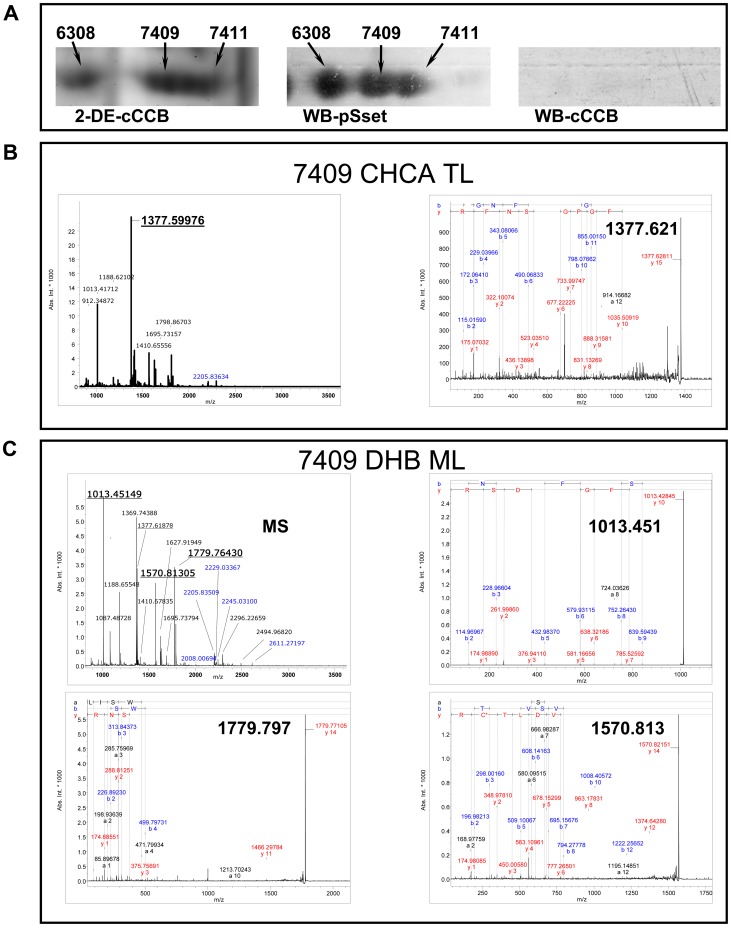
Identification of phosphoproteins with the pSset of MAbs and by MALDI-TOF mass spectrometry using two different matrix layer sample preparation methods. Two different MALDI sample preparation methods in combination enabled reliable identification of proteins. **Panel A:** In 2-DE-cCBB stained gels spot 7409 and 7411 are not well separated but WB with the pSset MAbs resolved three distinct spots. **Panel B:** With the CHCA TL sample matrix preparation the heterogeneous nuclear ribonucleoproteins A2/B1 (O88569) was identified by MALDI-MS and confirmed by MS/MS (Panel B, right spectra). **Panel C:** When the same spots were analyzed with the DHB ML sample preparation method glyceraldehyde-3-phosphate dehydrogenase (P16858) was additionally identified by MALDI-MS (upper panel C, left spectra marked as MS). Then, single protonated peptides were selected from the spectrum as precursor ions for MALDI-MS/MS analysis, i.e. 1013.451, 1779,797 and 1570, 813 m/z. Depicted are three individual MS/MS spectra of the selected precursor ions to confirm the identity of the protein.

### Animal Care

Mice C57BL/6 were maintained under specific pathogen free conditions in the facilities of the center and housed in Makrolon® Type III cages. Drinking water and food (V1124-000, SSNIFF, The Netherlands) were given *ad libitum*. The temperature and relative humidity were 22±2°C and 40–70%, respectively and a 12-h day and night cycle was used. Mice were anesthetized with CO_2_ and sacrificed. After surgical removal the lung was washed with PBS containing protease and phosphatases inhibitors until free of blood.

**Figure 4:**
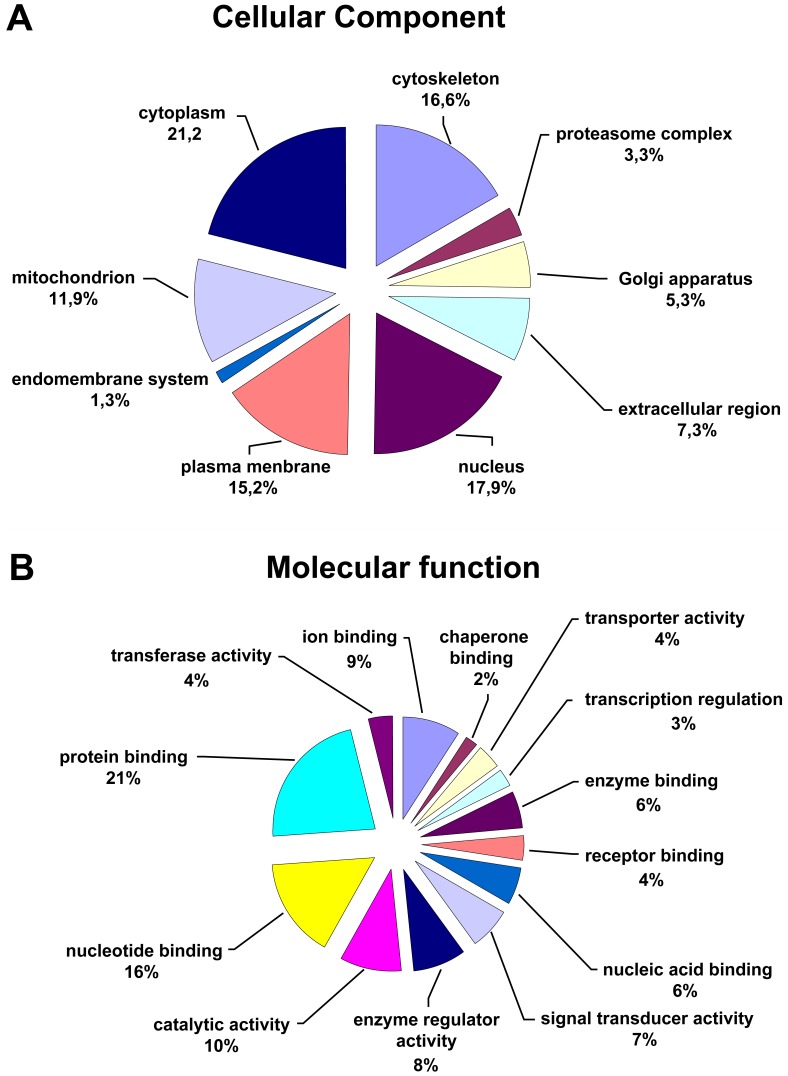
Distribution of identified proteins. Identified proteins were assigned on the basis of information provided by GO lists downloaded from PIR (http://pir.georgetown.edu/).to (a) cellular component or (b) molecular function.

### Materials

The following materials were obtained from Bruker Daltonics (Bremen, Germany): α-cyano-4-hydroxycinnamic acid (CHCA), 2,5-dihydroxybenzoic acid (DHB) and MALDI pre-structured sample support (AnchorChip™ 384/600). Sequencing grade modified trypsin was bought from Promega Co. (Madison, WI). Protease inhibitor kit III and Phosphatase inhibitor kit II were purchased from Merck (Darmstadt, Germany). The 4G10 and MPM-2 monoclonal antibodies were from Millipore (Billerica, MA), all other antibodies were obtained from ALEXIS (Enzo, Lörrach, Germany). The IPG pH 3-11 strips and buffer solution were obtained from GE Healthcare (München, Germany). All chemicals and reagents were of the highest grade available.

**Table 1:** Identified phosphopeptide by MALDI tandem MS using DHB ML sample preparation.

Protein Entry Name	Accession	Sequence	Start end	Meas. mass	Ion Score	Miss	MW
Angiotensin-converting enzyme	P09470	R.GPQFG**S**EVELR.H	1300–1310	1298.57765	63	0	151.888
Calnexin precursor	P35564	K.AEEDEILNR**S**PR.N	573–584	1508.67402	39	1	67.733
Advanced glycosylation end product-specific receptor	Q62151	R.KAPE**S**QEDEEER.A	373–384	1526.60058	60	1	43.068
Nascent polypeptide-associated complex alpha subunit	P70670	R.**S**V**T**DPA**M**APRTAK.N	588–600	1600.58786	20	1	221.603
Matrix-remodeling-associated protein 7	Q9CZH7	R.VAEPEE**S**EAEEPAAEGR.Q	73–89	1879.75921	82	0	19.516
Tensin	gi|226437589	R.**S**QSFPDVEPQLPQAPTR.G	790–806	1976.91134	56	0	203.317
Arginase-1	Q61176	-.**MSS**KPK**S**LEIIGAPFSK.G	1–17	2075.89248	35	0	34.999
60S acidic ribosomal protein	P99027	K.KEE**S**EE**S**DDD**M**GFGLFD.-	99–115	2125.68687	40	1	11.651
60S acidic ribosomal protein P1	P47955	K.KEE**S**EE**S**EDD**M**GFGLFD.-	98–114	2139.70251	40	1	11.610
Myosin phosphatase Rho-interacting protein	P97434	R.AEEQLPPLLSPP**S**PSTPHSR.R	280–299	2220.06959	27	0	117.357
Alpha-1 catenin	P26231	R.TPEELDD**S**DFETEDFDVR.S	634–651	2238.85983	127	0	100.896
Membrane-associated progesterone receptor component	O55022	K.EGEEPTVY**S**DDEEPKDETAR.K	173–192	2375.93983	112	1	21.692
60S acidic ribosomal protein P0	P14869	K.AEAKEE**S**EE**S**DED**M**GFGLFD.-	298–317	2410.81924	45	1	34.408
Septin-2	P42208	K.IYHLPDAE**S**DEDEDFKEQTR.L	210–229	2517.04527	115	0	41.783
EH domain-containing protein	Q8BH64	R.GPDEAIEDGEEG**S**EDDAEWVVTK.D	426–448	2557.01375	112	1	50.135
Elongation factor 1-delta	P57776	K.GATPAEDDEDKDIDLFG**S**DEEEEDKEAAR.L	145–173	3276.32225	144	2	31.916
Serum deprivation-response protein	Q63918	R.GNNSAVG**S**NADLTIEEDEEEEPVALQQAQQVR.Y	356–387	3520.57109	135	0	46.806
		R.RGNNSAVG**S**NADLTIEEDEEEEPVALQQAQQVR.Y	355–387	3676.67219	105	1	
		K.SSPFKV**S**PLSFGR.K	287–299	1488.72465	53	1	

Phosphopeptides identified by MALDI-TOF-MS/MS from 50 µg of protein extracted from mouse lung and separated by SDS-PAGE.

S = phosphorylated serine determined by MALDI TOF-MS/MS. M = oxidized methionine.

**Figure 5:**
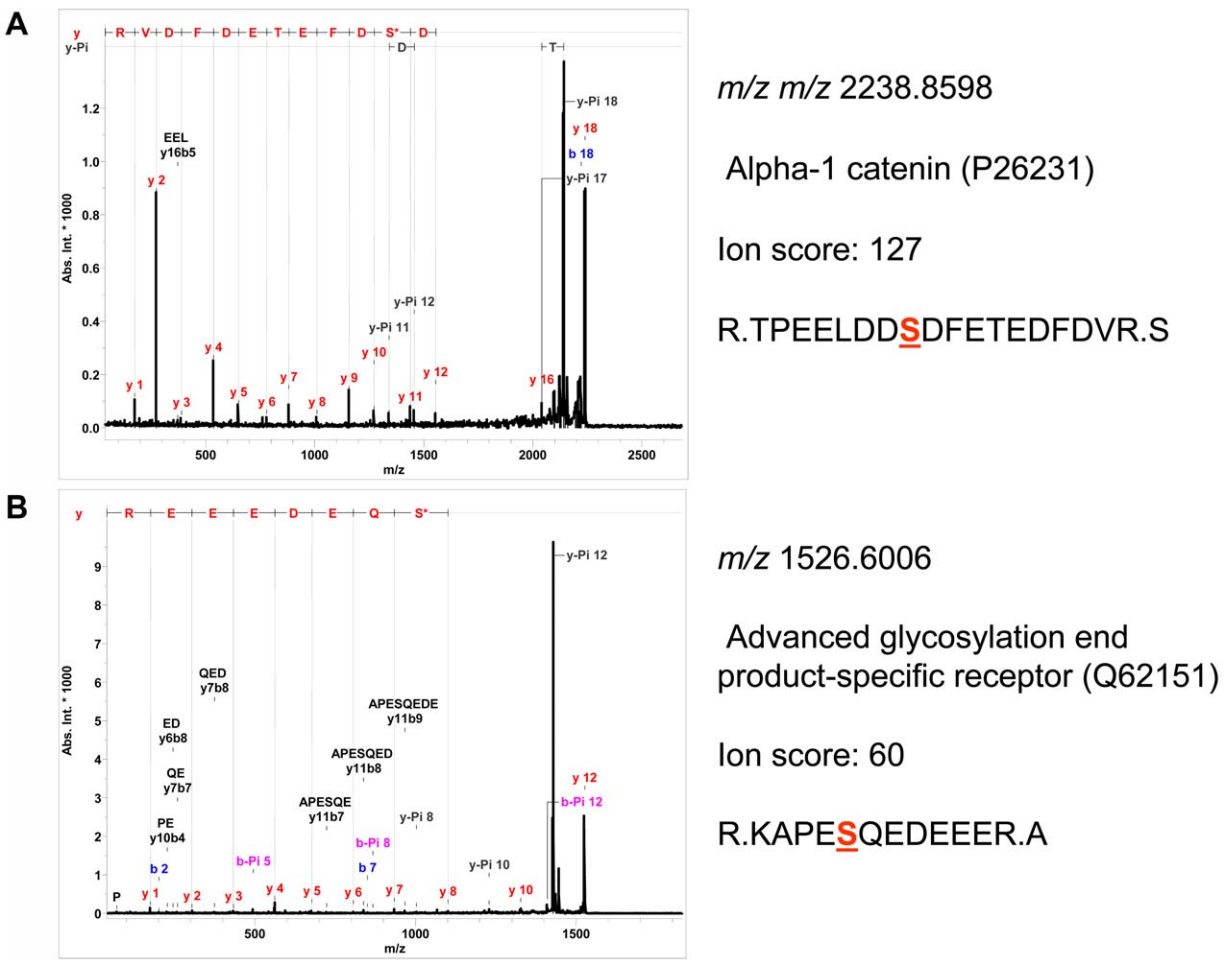
MALDI-TOF MS/MS of phosphopeptides. Characterization of phosphorylation sites of phosphorylated peptides by MALDI-TOF-MS/MS.

### Proteins Extraction

Whole lung tissue explants were washed twice with wash buffer (20 mM Tris-HCl, pH 7.4) containing phosphatase inhibitors and protease inhibitors. The tissue was disrupted with an ultrasound sonicator in lysis buffer (0,5% SDS, 100 mM DTT in 20 mMTris-HCl, pH 7.4) containing phosphatase inhibitors (diluted 1∶100) and protease inhibitors (diluted 1∶100). Then, proteins were precipitated with TCA/Acetone. After centrifugation at 20,000×g for 20 min at 4°C, the pellet was re-suspended in a buffer solution of 8 M Urea, 2 M Thiourea, 2% CHAPS, 2% ASB 14, 65 mM DTT, 5% IPG Buffer pH 3-11. The protein concentration was determined by the Bradford reagent (Sigma-Aldrich, Germany).

**Figure 6:**
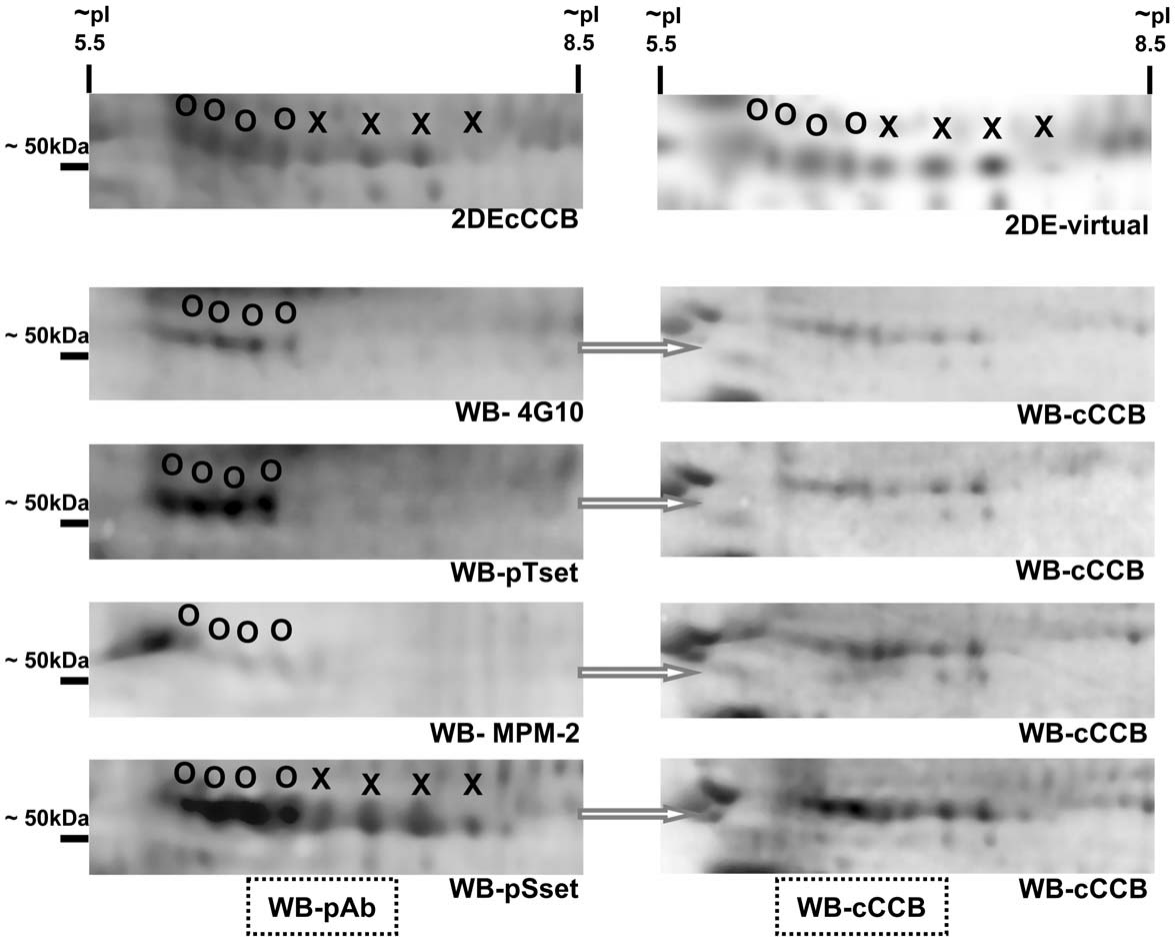
Specificity of the anti-phosphoproteins antibodies. The phosphoserine specific antibodies pSset recognized aldehyde dehydrogenase (P47738) and selenium-binding protein 1 (P17563), whereas the MPM-2, sYset and 4G10 identified the former only. O = spots identified as selenium-binding protein 1 (P17563); X = spots identified as aldehyde dehydrogenase (P47738). On the right side are depicted the same membrane after cCCB post staining.

### High-resolution 2-DE

250 µg of total tissue protein extracts were separated with 2-DE and gels were stained by colloidal Coomassie Brilliant Blue G-250 (cCBB) as described previously [25]. Phosphoproteins were detected by Western immunoblotting as described below.

### 1D SDS-PAGE

Proteins were loaded onto polyacrylamide gels [stacking: 4%T, 2.6%C (Bis); resolving gel 12%T, 2.6%C (Bis), 2% SDS] and ran in a Mini-PROTEAN III, (Bio-Rad, Hercules, CA) according to the manufacturer recommendations and stained with colloidal Coomassie Brilliant Blue (cCBB). Then, visible bands were excised and after in-gel protein digestion phophoproteins were enriched by metal oxide affinity chromatography as detailed below.

### In-gel Digestion of Proteins

CCB-stained protein-bands were removed from SDS-PAGE gels with a scalpel. Protein-spots from 2-DE gel were cut using EXQuest™ spot cutter (Bio-Rad, Hercules, CA). In-gel digestion was performed according to standard protocols [26] with minor modifications. The protein-bands and protein-spots were rehydrated in 30 µl and 16 µl of a digestion buffer, respectively containing 50 mM NH_4_HCO_3_ and 10 ng/µl of trypsin. The digestion was allowed to proceed at 37°C for 4 hours and was subsequently stopped finally with a 0.5% TFA solution.

### TiO_2_ Enrichment of Phosphopeptides

Enrichment of phosphorylated peptides was accomplished by metal oxide affinity chromatography (MOAC) with TiO_2_ beads on self-assembled disposable micro column (µ-MOAC-TiO_2_). For each sample to be purified, a micro purification column was packed into a 2–200 µl pipette tip (Eppendorf, Hamburg, Germany) using a small plug of glass microfiber filter (Whatman, Dassel, Germany) as a frit. The column consisted of a very small volume bed (∼1.5 mm thick bed stationary phase) of TiO_2_ beads (5µm; GL Sciences, Tokio, Japan). The µ-MOAC TiO_2_ were conditioned/activated with 2×30 µl of acetonitril, equilibrated with 2×30 µl of loading/wash (L/W) solution (50% acetonitrile, 1% TFA solution). Typically, 30 µl of tryptic digest solution were loaded onto the column and washed with 3×30 µl of LW solution. The phosphopeptides were eluted from the column with 3×10 µl of elution solution (5% NH_4_OH). The elutes were reduced to dryness in a rotor concentrator (Christ, Germany) and resuspended in a solution of 5% acetonitrile, 0.1% TFA suitable for MALDI-MS analysis.

### MALDI Matrix Sample Preparation Protocols

A CHCA thin-layer (TL) matrix was prepared and deposited on the AnchorChip™ (Bruker Daltonics) sample support as described previously [27]. Once dry, 1 µl of in-gel tryptic digest was deposited and thereafter re-crystallized “on-target” using 0.5 µl of re-crystallization solution (ethanol: acetone: TFA 0.1%, 6∶3:1). In addition, a DHB matrix layer (ML) was prepared and deposited on the AnchorChip™ sample support (Bruker Daltonics) using the conditions detailed in [27]. Without further modifications either 1 µl of in-gel tryptic digest or TiO_2_-MOAC enriched phosphopeptide extracts, were deposited to await MALDI-MS analysis.

### Mass Spectrometric Analysis

MALDI-MS experiments were carried out with an Ultraflex II MALDI-TOF/TOF mass spectrometer equipped with a SmartBeam™ laser and a LIFT-MS/MS facility. The instrument was operated in positive ion reflectron mode and an acceleration voltage of 25 keV. Typically, 400 spectra over a 600–4000 *m/z* mass range, acquired at 100Hz, were summed and externally calibrated. The MS/MS-CID spectra were obtained by use of the LIFT device for selection and fragmentation of the ions, acceleration voltage in the ion source was 8 kV, the Timed Ion Selector was set to 0.4% (relative to parent mass), and argon was used as collision gas (∼4–6×10–6 mbar). Resulting fragments were further accelerated in a second source by 19 kV and analyzed by a two-stage gridless reflectron. Typically, 600 shots were accumulated for the parent ion signal and 1000–2500 shots for the fragments. FlexControl™ 3.0, and FlexAnalysis™ 3.0 were used as instrument control and processing software (instrumentation and software from Bruker Daltonics, Bremen, Germany).

### Calibration

Spectra were externally calibrated using a peptide calibration standard that is a mixture of seven peptides (cod. # 22570, Bruker Daltonics). The mixture was spotted onto positions adjacent to the samples. Additional internal mass calibration of peptide mass maps was performed using trypsin autolysis products (*m/z* 842.51 and *m/z* 2211.10).

### Protein Identification

Automated MALDI-MS spectra acquisition, tandem MS/MS spectra selection and acquisition as well as protein identification were carried out with the Proteinscape software (Bruker Daltronics), using the SwissProt protein database and Mascot searches for variable modifications (v. 2.0, Matrix sciences; UK) software [28]. Sequence database searches were done using the following search parameters for MS and MS/MS data: all entries; mass tolerance: 100 ppm for parent ion; 0.9 Da for fragments, one missed cleavage site; cysteine residues modified with acrylamide and methionine oxidation as partial modification.

### Western Blot Analysis

After 2-DE separation, the proteins were electro-blotted onto PVDF membrane. The blotted membranes were rinsed with TBS buffer (100 mM NaCl, 10 mM Tris-HCl ph 7.5) and blocked with 3% BSA in TBS buffer. Thereafter, membranes were rinsed with TBS-T solution (0,1% Tween in TBS buffer) to remove excess of BSA and incubated with different anti-phosphoprotein specific monoclonal antibody (MAb) solutions (see Table S1) diluted as 1∶1000 with 3% BSA in TBS-T buffer for a minimum of 90 min to over night at 4°C. Then the membrane were washed 4 times with TBS-T solution, and incubated with horseradish peroxidase-goat anti-mouse conjugate polyclonal anti-IgG or anti-IgM antibodies, diluted as 1∶1000 in TBS-T containing 1% BSA for about 90 min. The protein-antibody complexes were visualized with an OPTI-4-CN kit (Biorad), air dried. Subsequently, reference points were added and images acquired. Finally, dry membranes were immersed in cCCB-post-staining solution [5% Al_2_(SO_4_)_3_ 2% H_3_PO_4_, 25% ethanol, 0.02% Coomassie G-250] for 15 minutes, then washed with water, and new images were acquired. Each sample was run in triplicate, reprehensive results are presented.

### Image Acquisition and Analysis

The images of cCCB stained gel, immuno-developed and cCCB-post-stained PVDF membranes were acquired with an Expression 10000 XL scanner (Epson, Germany) at 16 bit and 50 µm/pixel. Prior to image acquisition of the immuno-developed PVDF membranes, 8–12 dots as reference point were added manually on the boundary region and in the middle of membrane with a marker pen. Image analysis was done with the software PDQuest v 8.0.1 (Bio-Rad), and the alignment and matching process was supervised.

### Prediction and Classification of Phosphoproteins and Phosphorylation Sites

The NetPhos 2.0 server (http://www.cbs.dtu.dk/services/NetPhos/) [29] was used to predict the number and position of potential protein phosphorylation sites. Database searches within PhosphoSitePlus® (PSP) (http://www.phosphosite.org/) [30], Phospho.ELM 9.0 (http://phospho.elm.eu.org/) [31] and UniProt (http://www.uniprot.org/) [32] were additionally carried out to gain information about the exact position of known phosphorylated sites. Scansite 2.0 (http://scansite.mit.edu) [33] was used to predict candidate Ser/Thr- or Tyr-kinases. Here, motive searches of specific amino acid sequence of phosphorylated protein helped to identify candidate kinases responsible for the phosphorylation. Finally, gene ontology (GO) lists were downloaded using the protein information resource (PIR) (http://pir.georgetown.edu/) [34]. Each protein was classified with respect to its cellular component and molecular function using GO annotation.

### A Combined 2-DE-Western Blotting Approach

To screen for phosphorylated proteins a combination of different techniques and technologies were employed, i.e. (1) 2-DE, (2) 2-DE-Western blotting with antibodies recognising phosphorylated proteins, (3) computer assisted image analysis and processing, (4) MALDI-TOF tandem mass spectrometry (MS) with automated spectra acquisition and analysis. These technologies have been put together as depicted in Figure 1. A detailed description of the procedure is given below.

Mice were sacrified and lungs were removed and washed free of blood. Then the lung tissue was disrupted with a sonicator and the proteins extracted in the presence of detergents (SDS), reducing agents (DTT), protease and phosphatase inhibitors. Preparative gels were loaded with 250 µg of tissue lysate proteins and separated by 2-DE, then stained with Colloidal Coomassie G-250 (cCCB) and the image acquired to generate a reference image of the total protein extract (2-DE-cCBB). Furthermore, analytical gels were prepared by loading 40 µg of proteins that were subsequently separated by 2-DE, transferred onto PVDF membrane followed by immuno-detection using anti-phosphorylated proteins specific monoclonal antibodies (WB-pAb). With a marker pen (rapidograph®, rotring, Germany) several dots were added on the PVDF membrane and used as landmarks for membrane images that were superimposed (see below) and the image was acquired. Thereafter, the membrane was post-stained using cCCB, to visualize total proteins blotted, and a new image was acquired (WB-cCCB). Using the dots as landmarks the WB-pAb and the WB-cCCB images were merged by the image analysis software as superimposed and matched by creating an image showing the phosphoproteins and the total proteins together (WB-Virtual). Eventually, the 2-DE-CBB image was superimposed and matched to the WB-Virtual image using the CCB post-stained protein spot on the membrane as landmarks, to unravel phosphorylated proteins on the 2-DE-cCBB gel. Protein spot landmarks were selected in such a way to be evenly distribution through the gel. This allowed the software algorithm to compensate local gel distortions. Protein spots on the 2-DE-cCBB gel showed the same electrophoretic mobility as the immunodetected proteins in the membrane were excised from the gel, subjected to in-gel tryptic digestion and identified by MALDI-TOF/TOF-MS. Each immunoblot was run in parallel with one or more 2-DE-cCBB gels, in order to be matched with. At the same time, the protein spots used as landmark for the matching process were also excised and identified on every different 2-DE-cCBB gel to confirm the accuracy of the matching procedure.

## Results and Discussion

D*e-novo* phosphoproteomic analysis from limited sample material is challenging. To demonstrate the utility of the developed method lung tissue of mice was analyzed and protein extracts were separated by 2-D PAGE. Representative 2-DE maps of tissue lysates and immunoblots are shown in Figure 2. On average 450±30 protein spots per gel were visualized by colloidal CCB staining (cCCB) at a pI range of 3–11. The identified proteins and other relevant information are given in Table S1.

### 2-DE Phosphoprotein Analysis of Tissue Lysates

Successful research into protein phosphorylation requires both a selective separation and enrichment procedure and a reliable method for the detection, identification and characterization of phosphoproteins. When phosphorylated proteins are detected by antibodies, it is important to realise that each antibody (Ab) has its own unique properties regarding sensitivity and specificity. In addition, factors such as background from contaminating proteins and nonspecific reactivity of the antibodies toward unrelated proteins could influence the performance as well. In the present study, the employed monoclonal antibodies (MAbs) were validated previously for their usefulness in the enrichment of phosphorylated proteins and were confirmed by MS identification, therefore demonstrating specificity and selectivity [14]. Supplementary Table S2 informs on the properties and the combination of antibodies used for the search of pulmonary phosphoproteins. Notably, the various MAb were produced with slightly different antigens, which might influence the specificity and binding behavior of the Ab. Alongside the immunoblotting of phosphoproteins their identification by MS and the subsequent submission of identified sequences to software predictor and database searches of already known literature information confirmed the reliability of the identified data as phoshorylated proteins. Consequently, detection of phosphoproteins with specific Ab and their identification by high performance MALDI-TOF-MS/MS was a simple and fast method. Moreover, to increase the sequence coverage and for comprehensive analysis, the trypsin digested protein spots were analyzed by two different sample preparations, i.e. the CHCA thin layer (TL) and DHB matrix layer (ML). Then, MS/MS data were used to confirm identified proteins. Once identified, bioinformatics was used to predict the potential protein phosphorylation sites, and database searches provided information about the exact position of known phosphorylated sites. In addition to the immunoblotting of phosphoproteins, extracted proteins of lung tissue were also separated by one dimensional SDS-PAGE, and after in-gel digestion of individual bands phosphopeptides were enriched with self-made TiO_2_ micro column. The phosphorylation sites were then identified and characterized by MALDI-TOF-MS/MS.

### Detection and Identification of Phosphorylated Proteins with Anti-phosphoserine Antibodies

For the detection of serine phosphorylated proteins, a set of 5 different anti-phosphoserines (pSset) and the MPM-2 MAbs were used (Supplementary Table S2). After development on PVDF membranes (Figure 2) with the pSset of monoclonal antibodies, the corresponding spots were excised from the 2-DE cCBB stained gel, in-gel digested with trypsin to overall yield 149 unique proteins (Table S1). In Figure 3 an example of the specificity of the pSset of MAbs is given. Three distinct spots detected in the WB with the pSset MAbs had two corresponding spots in the 2-DE-cCBB stained gel. Importantly, with the CHCA TL sample matrix preparation the heterogeneous nuclear ribonucleoproteins A2/B1 (O88569) was identified and confirmed by MS/MS. However, when the digests of the same spots were analyzed by the DHB ML sample preparation Glyceraldehyde-3-phosphate dehydrogenase (P16858) was additionally identified and confirmed by MS/MS. Therefore, a careful comparison of the spot in the developed immunoblot of the 2-DE-cCBB gel and the visual inspection of regions in which the spots appeared needed to be confirmed. Likewise co-migrated and adjacent spots were examined. Specifically, the use of two different MALDI sample preparation methods and the acquisition of MS/MS spectra in an automated mode enabled reliable identification of phosphoproteins. The pSset of MAbs facilitated the MS identification of a large number of kinases including the SRC kinase (P05480), inhibitor of nuclear factor kappa-B kinase subunit beta (O88351), integrin-linked protein kinase (O55222) and serine-protein kinase ATM (Q62388) mitogen-activated protein kinase 3 (Q63844). For spots commonly identified by immunoblotting and by 2-DE MALDI MS/MS experiments were additionally performed (Table S1) With the MPM-2 MAb 43 unique proteins were detected by WB. Despite the overall reduced number of proteins detected with the this MAb unique proteins (n = 6) were identified and had not been detected by the pSmix Abs most likely as a result of differences in specificity of epitop recognition. One of these proteins is the heat shock protein HSP 90-alpha (HS90A). This chaperone is essential for proper protein folding, stabilization and trafficking of an expanding list of proteins, but appears to be critical for a variety of signal transduction pathways as well through an interaction with a wide range of transcription factors and protein kinases. Moreover, inhibition of HS90A activity is pursued for the treatment of various kinds of cancer including lung cancer [35], [36]. Of note, the use of the software Scansite predicted HS90A as a substrate for Fgr Kinase, Src Kinase, Lck Kinase, Akt Kinase, Casein Kinase 2 and the Erk D-domain and database search as with Phospho.ELM and PhosphoSitePlus confirmed the phosphorylation of serine, threonine and tyrosine in mouse and humans based on other experimental data.

### Detection and Identification of Phosphorylated Proteins with Anti-phosphotyrosine Antibodies

For the detection of tyrosine phosphorylated proteins a set of 4 different monoclonal anti phosphotyrosine (pYset) and the 4G10 MAb were used (Table S2). With the pYmix MAbs (Figure 2) 54 unique proteins were detected and the corresponding spots on 2-DE-cCBB stained gels were excised. Thereafter trypic digestes were prepared and analysed by MALDI-MS. With the 4G10 MAb 40 phosphoproteins were detected, however 6 were unique when compared with the immunoblots obtained with the pYmix of MAbs. (Figure 2).

### Bioinformatic Analysis of Phosphoproteins

Functional annotation of identified phosphoproteins was achieved by categorizing the proteins using GO terms. Figure 4A and B depicts a pie-chart distribution of the identified proteins catalogued according to the cellular component or molecular function (Supplementary Table S3). Out of the 160 phosphoproteins identified their distribution was assigned to 21.2% into cytoplasm, 17.9% in the nucleus, 11.9% in the mitochondria, 16.6% in the cytoskeleton, 15.2% in the plasma membrane and 7.3% as extracellular. Such a distribution of proteins provided further evidence of the versatile character of the developed assay in the search for novel phosphoproteins. The molecular functions of the annotated proteins are consistent with the activity of phosphoproteins in biological processes including protein binding (22.2%), nucleotide binding (15.7%), catalytic activity (9.8%), enzyme regulation (8.5%), enzyme binding (5.9%) and signal transduction (6.5%). Since lung served as a source of tissue material, it is not surprising that the functions of most identified phosphoproteins are related to respiratory biology.

The PhosphoSitePlus®, Phospho.ELM 9.0 and the UniProt database were used to search for the exact position of known phosphorylation sites based on the available literature. Furthermore, putative phosphorylation sites were searched with different software packages. With Netphos 2.0, an output score of 0.5 was employed as cutoff to ensure that the obtained result is a *bona fide* phosphorylation site. Notably, for all of the 160 identified proteins several phosphorylation sites were predicted. Each phosphoprotein is further analyzed with Scansite 2.0 with high and medium stringency to predict the kinase and phosphorylation-dependent binding motifs. Potential binding sites for the Erk D-domain, PDK1 Binding, DNA PK, ATM kinase and casein kinase are most commonly predicted; casein kinase 1 and 2, PKC epsilon and zeta sites are also found frequently. Thus, out of 160 proteins analyzed 106 (66.2%) are already validated phosphoproteins based on the available literature, 26 proteins (16.2%) were predicted in mouse but validated as phosphoproteins either in humans or in rats, and 30 (18.8%) are only predicted to be phosphorylated but enharbour a kinase docking domain (Supplementary Table S1). In all, a total of 130 phosphoproteins (81.25%) are validated; this documents the usefulness of the developed assay.

### Enrichment of Phosphoproteins by MOAC and *de novo* Identification of Phosphoryation Sites by MALDI-TOF-MS/MS

Several reports describe the use of titanium in metal oxide affinity chromatography (MOAC-TiO_2_) for the successful enrichment of phosphorylated peptides. We found most of these protocols not useful for our sample preparation due to the elevated concentration of DHB in the elution solution and the increased backpressure obtained [20]. Therefore, we developed a disposable micro-column (µ-MOAC-TiO_2_) in which inert glass microfiber was used as a frit and the composition of the elution solution was modified. To further characterize the position of the phosphorylation site, trypsin digests of immunoreactive spots were enriched by µ-MOAC-TiO_2_ and subsequently applied to the pre-structured sample support for MS analysis. The obtained MALDI-TOF-MS spectra were analyzed for the presence of phosphorylated peptide signals. None of the digested proteins gave acceptable signals of phosphorylated peptides (data not shown). Considering the low stoichiometry of phosphoporylation the total amount of peptides in the 2-DE spots digests was simply insufficient for an identification of phosphopeptides. Tissue extracted proteins were therefore separated by one-dimensional SDS-PAGE and enriched as follows: 50 µg of lung extracted proteins were loaded on SDS-PAGE; the lane was cut into 15 regions. The digested proteins from each region were enriched using µ-MOAC-TiO_2_ and analyzed by MALDI-TOF-MS. Each peptide signal presented in the MS spectrum was subjected to MS/MS fragmentation. With this approach additional 17 proteins and 19 phosphorylated peptides were identified and characterized by MALDI-TOF-MS/MS. Specifically, 14 peptides contained a single pSer residue, 3 peptides contained two and 1 peptide contained three pSer residues. Moreover, one protein with a single pSer residue also contained pThre residues as reported in Table 1. The yielded spectra contained the characteristic neutral loss of ions at -98 and -80 from the molecular ion as result of β-elimination of H_3_PO_4_. The high quality of spectra enabled detection of copious signal from the peptide back bone (y- and b- series) leading to an unambiguous characterization of the phosphorylation site(s), and in some cases the complete peptide sequence was observed. In addition, peptide ions of masses of>2000 Da were detected to yield highly informative and structurally relevant fragment ions as shown in Figure 5A for the peptide R.TPEELDD**S**DFETEDFDVR.S of alpha-1 catenin (P26231). In Figure 5B the MALDI-TOF-MS/MS spectra of peptide *m/z* 1526.5210 that corresponds to the peptide R.KAPE**S**QEDEEER.A of the advanced glycosylation end product-specific receptor protein (RAGE_MOUSE) is depicted. The fragmentation pattern allowed not only an unambiguous identification of the peptide through the main fragmentation signal but the identification of the Ser 377 as the definitive phosphorylation site. Noteworthy, this protein is described as phosphorylated on threonine 271 but not as phosphorylated on serine (377) in mouse lung. While TiO_2_ has been widely used for the enrichment of phosphorylated peptides, none of the presented works demonstrated the feasibility of such enrichment for MALDI-MS for the specific characterization of phosphorylation sites in tissue extracts. Importantly, the copious neutral loss in MS/MS spectra shadows the signal of the backbone peptide. With the CHCA TL sample preparation method a reduced number of phosphorylated peptides, copious matrix clusters, and increased fragmentation rendered data analysis impossible (data not shown). However, the DHB ML sample preparation method delivered an improved homogeneity and increased backbone peptide fragments signal in MS/MS experiments when the matrix was doped with diammonium hydrogen phosphate. Therefore, the ML sample preparation in combination with µ-MOAC-TiO_2_ enrichment allowed the successful fragmentation and characterization of novel phosphorylation sites.

Taken collectively, the experimental strategy consisted of two approaches in the search of phosphoproteins. The first approach is based on immunoblots with anti-phosphorylated protein MAbs, while the second approach is based on enrichment of phosphopetides from tissue extracts with TiO_2_. The immunoblot procedure does not always define all the possible residues subjected to posttranslational modifications. As shown in Figure 6, the phosphoserine specific MAbs pSset recognized aldehyde dehydrogenase (P47738) and selenium-binding protein 1 (P17563), but with the MAbs 4G10, MPM-2 and pYset only one of these proteins were detected. These two proteins are phosphorylated at different sites as reported elsewhere. Thus, by use of several anti-phosphoprotein Abs with different specificity in the recognition of epitopes a more comprehensive characterization of phosphoproteins can be achieved. Furthermore, the identified proteins enharbour kinases binding motifs as suggested by various bioinformatic sequence analysis. Moreover, 66% of all the detected proteins have been reported to be phosphorylated at sites identified in the present study and appeared to be conserved in human, rat and mouse.

### Conclusions

An antibody based rapid screening method for detection and *de novo* identification of phosphoproteins was developed and applied to lung tissue for an identification of pulmonary phosphoproteins. The facile use of MALDI-TOF-MS/MS for the characterization of phosphopeptides is therefore demonstrated. The assay can readily be applied to any tissue in the search for phosphoproteins and to obtain information about on/off states with regard to protein phosphorylation. In the same way it would be possible to study the effects of drug treatment with kinase inhibitors on the phosphoproteome. The proposed assay provided the capability to qualify protein phosphorylation status on a systematic scale and therefore can be employed in biomedical research.

## Supporting Information

Table S1 Summary of sequence specific phosphoresidues of identified proteins and bioinformatic prediction of kinases involved.
(XLS)

Table S2 Monoclonal antibodies and their properties.
(DOC)

Table S3 GO annotations of identified proteins.
(XLS)
