# Supplementary material for: A Rapid Screening Assay to Search for Phosphorylated Proteins in Tissue Extracts
Source: PLoS One. 2012 Nov 15;7(11):e50025. doi: 10.1371/journal.pone.0050025 (PMC3499474; doi:10.1371/journal.pone.0050025)
Supplement: Table S2 — Monoclonal antibodies and their properties. (DOC) [file pone.0050025.s002.doc]

**Table S2: Monoclonal antibodies and their properties**

| *Name* | *Purity details* | *Host/Type/Isotype* | *Immunogen* | *Application tested* | *Specificity* | *Control used in 2-DE WB* | *Used as:* | *Supplier/Cat.#* |
| --- | --- | --- | --- | --- | --- | --- | --- | --- |
| **Anti-Phosphotyrosine, Clone 4G10®** | IgG2bκ mouse monocolonal antibody produced in vitro by mouse-mouse hybridoma 4G10® (FOX-NY [NS-1 derivative] myeloma x spleen cells). | Mouse/  M/  IgG2bκ | Phosphotyramine-KLH | IP, WB, IC, IH | Recognizes tyrosine-phosphorylated proteins from all species. | EGF-stimulated A431 whole cell lysate  (deliverd) | **4G10** | Upstate(Milllipore)/  05-321 |
| **Phosphotyrosine, mAb, 2C8** | Purified from serum-free cell culture supernatant by subsequent thiophilic adsorption and size exclusion chromatography. | Mouse/  M/  IgG1 | Phosphotyrosine containing peptides. | WB | Epitope: GpYY... Recognizes a broad range of tyrosine-phosphorylated proteins. |  | **pYset** | Enzo Life Sciences(Alexis)/  ALX-804-171 |
| **Phosphotyrosine, mAb, 3B12** | =2C8 | Mouse/  M/  IgG1 | Phosphotyrosine containing peptides. | WB, IH | Recognizes phosphotyrosine in the context of the surrounding aas. |  | **pYset** | Enzo Life Sciences(Alexis)/  ALX-804-172 |
| **Phosphotyrosine, mAb, 9H8** | =2C8 | Mouse/  M/  IgG1 | Phosphotyrosine containing peptides. | WB | Epitope: .RGpYVP...Recognizes phosphotyrosine in the context of the surrounding amino acids, tolerating positively charged amino acids N-terminal to phosphotyrosine. |  | **pYset** | Enzo Life Sciences(Alexis)/  ALX-804-173 |
| **Phosphotyrosine, mAb, 16F4** | =2C8. | Mouse/  M/  IgG1 | Phosphotyrosine containing peptides. | WB | Epitope: .DIpYAE...Recognizes phosphotyrosine in the context of the surrounding amino acids, tolerating hydrophobic amino acids directly neighbouring the phosphotyrosine. |  | **pYset** | Enzo Life Sciences(Alexis)/  ALX-804-174 |

| *Name* | *Purity details* | *Host/Type/Isotype* | *Immunogen* | *Application tested* | *Specificity* | *Positive Ctrl in 2-DE WB* | *Used as:* | *Supplier/Cat.#* |
| --- | --- | --- | --- | --- | --- | --- | --- | --- |
| **Anti-phospho-Ser/Thr-Pro, MPM-2 (Mitotic protein monoclonal #2)** | Protein G Purified immunoglobulin. | Mouse IgG1 | Mitotic human HeLa cell cytosolic lysate | IP, WB, ELISA, IH | Recognizes phosphorylated serines or threonines when followed by a proline. Binds to a phospho amino acid-containing LTPLK and FTPLQ domains. | - | **MPM-2** | Upstate(Milllipore)/  05-368 |
| **Phosphoserine, mAb, 1C8** | Purified from serum-free cell culture supernatant by subsequent thiophilic adsorption and size exclusion chromatography. | Mouse/  M/  IgM | Phosphoserine containing peptides. | WB | Recognizes a broad range of serine-phosphorylated proteins in crude cell extracts, interacting with phosphoserine surrounded by positive or neutral aa (e.g. substrates of PKA, PKB, PKC, PKG etc.). Specificity has been determined by epitope mapping using degenerated phosphopeptide libraries. | Phosphoproteins from rabit muscle isolated by affinity chromatography on a Fe ^3+^ IDA colum | **pSset** | Enzo Life Sciences(Alexis)/  ALX-804-162 |
| **Phosphoserine, mAb, 4A3** | =1C8 | Mouse/  M/  IgM | Phosphoserine containing peptides. | WB | =1C8. |  | **pSset** | Enzo Life Sciences(Alexis)/  ALX-804-163 |
| **Phosphoserine, mAb, 4A9** | =1C8 | Mouse/  M/  IgM | Phosphoserine containing peptides. | WB | =1C8. |  | **pSset** | Enzo Life Sciences(Alexis)/  ALX-804-164 |
| **Phosphoserine, mAb, 4H4** | =1C8 | Mouse/  M/  IgM | Phosphoserine containing peptides. | WB | =1C8. |  | **pSset** | Enzo Life Sciences(Alexis)/  ALX-804-165 |
| **Phosphoserine, mAb, 16B4** | =1C8 | Mouse/  M/  IgM | Phosphoserine containing peptides. | WB | Recognizes a broad range of serine-phosphorylated proteins in crude cell extracts, interacting with pSK and pSP motifs, thus specifically recognizing substrates of MAP/SAP kinases and CDC kinases. |  | **pSset** | Enzo Life Sciences(Alexis)/  ALX-804-167 |

Abbreviation: monoclonal: M, western blot: WB, immunoprecipitation: IP, immunohistochemistry: IH, immunocytochemistry: IC, Phosphotyrosine mix: pYmix
